# Smart Speaker–Based Applications to Support Social Connectedness in Older Adult Residents in Affordable Housing: User-Centered Design Study

**DOI:** 10.2196/90053

**Published:** 2026-07-07

**Authors:** Jane Chung, Natalie Mansion, Tracey Gendron, Rachel E Wood, George Demiris

**Affiliations:** 1 Nell Hodgson Woodruff School of Nursing, Emory University, 57 Executive Park Dr NE, Atlanta, GA, 30329, United States, 1 404-544-9589; 2 School of Nursing, Virginia Commonwealth University, Richmond, VA, United States; 3 Department of Gerontology, College of Health Professions, Virginia Commonwealth University, Richmond, VA, United States; 4 Department of Nursing, Longwood University, Farmville, VA, United States; 5 School of Nursing, University of Pennsylvania, Philadelphia, PA, United States

**Keywords:** social connectedness, loneliness, social isolation, smart speakers, disparities, user-centered design, community-dwelling older adults, focus groups

## Abstract

**Background:**

Older adults in affordable housing face heightened risks of social isolation and loneliness due to limited social networks, transportation barriers, chronic conditions, and inadequate technology access. Smart speakers offer potential for enhancing social connectedness in this underserved population, yet technology interventions are rarely designed with meaningful input from older adults themselves. User-centered design (UCD) approaches can address this gap by engaging end users throughout the development process to ensure technology solutions align with their needs and living contexts.

**Objective:**

This study aimed to engage older adults in affordable housing in an iterative UCD process to develop prototype scenarios for smart speaker–based applications that promote social connectedness while addressing safety, community-building, and wellness needs.

**Methods:**

We conducted a 3-stage UCD study with 29 older adults (mean age 70, SD 6.8 years; 23/29, 79% African American; 20/29, 69% high school education or less) living alone in affordable housing between April 2021 and April 2022. Stage 1 included 5 focus groups (n=25) combining needs assessment discussions with rapid brainstorming activities. Stage 2 involved research team synthesis of focus group transcripts and brainstorming data to create a Design Strategies Map, development of initial prototype scenarios, 5 evaluation focus groups (n=18) to gather feedback, and iterative scenario refinement. Stage 3 comprised 4 validation focus groups (n=17) to assess refined scenarios and identify implementation recommendations. Participants included both smart speaker users (n=13) and nonusers (n=16). Data were analyzed using thematic analysis for needs assessment, content analysis for brainstorming ideas and feedback, and matrix analysis for systematic comparison across scenarios.

**Results:**

Participants generated 153 ideas for smart speaker use, with Health and Safety and Daily Assistance being the most frequent categories. Analysis revealed that social connection needs were inseparable from safety concerns related to living alone. Through iterative co-design, we developed 7 prototype scenarios across 4 functional categories: Checking-In (peer and management safety verification with privacy controls), Social Companion (conversational artificial intelligence–based companionship and emotional support), Community Involvement (virtual bulletin boards and activity coordination), and Wellness Check (system-initiated monitoring of activity and behavioral patterns as health indicators with user-controlled interventions). Participants emphasized requirements for personalization, opt-in/opt-out controls, “Do-Not-Disturb” functionality, and safeguards preventing replacement of human connection.

**Conclusions:**

Older adults in affordable housing engaged in technology design and provided valuable insights that challenge assumptions about their needs and preferences. The prototype scenarios addressed the dual imperatives of social connection and safety while living alone, offering a foundation for developing technology-based applications tailored to underserved populations. Implementation should prioritize user control, privacy protection, and human-in-the-loop design ensuring that technology facilitates rather than replaces human connection and community programming, alongside consideration of user characteristics to build trust and ensure effective, sustained use of the intended technology platform.

## Introduction

### Background

Research has repeatedly demonstrated the critical importance of social connectedness in older age. Social isolation and loneliness, the main constructs of social disconnectedness, are prevalent in Americans aged 65 years and older, with prevalence rates approaching nearly 25% for the former [1] and ranging from 17% to 57% for the latter [2-4]. Social isolation is objectively defined as the lack of social contact, communications, and relationships [1,5], while loneliness refers to a subjective feeling resulting from a perceived deficit in the quantity or quality of meaningful social relationships [6]. Older adults who are socially isolated or feel lonely exhibit a wide range of negative health outcomes, including depression, cardiovascular diseases, poor cognitive function, reduced quality of life, and increased mortality [2,5,7,8].

Social isolation and loneliness are pressing concerns for older adults living in affordable housing (hereafter referred to as “residents”) because they have limited social networks, transportation options, and access to information and communication technology (ICT) [9]. Chronic health conditions and disabilities are also common in this population [10,11], which affect their ability to establish and maintain meaningful social interactions. Also, affordable housing, particularly in high-rise structures, may lack communal areas [12] or discourage residents from inviting others over due to suboptimal building conditions [13]. The complex interplay of health, housing conditions, and social and financial factors may contribute to elevated levels of social isolation and loneliness among residents [14]. Innovative approaches are necessary to increase meaningful social connections and reduce loneliness in this population. It is recognized that there is no “one-size-fits-all” approach to address this issue and there is a need to design interventions customized to meet specific groups or the varying levels of social isolation and loneliness they may experience [15].

A notable gap exists in technology-based interventions tailored to enhance social connectedness among residents. While face-to-face programs and services for improving social connections are available for older adults (eg, community senior centers and senior cafés), these may be inaccessible to older adults residing alone in affordable housing for multiple reasons mentioned previously. The growing interest in voice-operated smart speakers (eg, Amazon Echo and Google Home) has increased an exploration into how this technology can enhance social connectedness in community-dwelling older adults [16-18]. The hands-free voice control may improve accessibility and usability for people with vision impairment, mobility limitation, or poor dexterity. Artificial intelligence (AI)–based voice agents (eg, Amazon Alexa) on smart speakers have the potential to help older adults in limited-resource settings feel less lonely and engage more in social activities, for example, by providing easy access to communicate with others. Studies provide preliminary evidence that older adults enjoy social health benefits from interactions with an AI voice agent, such as a source of companionship reducing loneliness and improving mental health [18,19].

Despite the potential benefits of smart speakers, unique challenges to their adoption and use may arise, particularly when the needs of target end users are overlooked or inadequately addressed. Moreover, historically marginalized communities, including low-income older adults and racial or ethnic minority groups, still face inequity in ICT access as well as challenges in its adoption and use [20-22]. Low ICT adoption and utilization are attributed to various barriers, including technology costs, internet accessibility, functional impairments, low literacy, lack of self-efficacy, privacy concerns, and mistrust of the technology [23-28]. Recognizing the unique needs of users and the factors influencing acceptance and usability within the user’s living context is critical for implementing technology-based interventions. This can be accomplished through user-centered design (UCD), which involves exploring user needs, preferences, and technology requirements; engaging users throughout the design process; and iteratively developing technology prototypes [29].

### Aims

The purpose of the study was to engage older adult residents living alone in affordable housing in an iterative UCD process to develop prototype ideas of innovative solutions leveraging smart speaker technology for social connectedness. Through a 3-stage co-design process, we sought to (1) understand the social connection needs and technology preferences of residents, (2) collaboratively develop and refine prototype scenarios that address these needs, and (3) identify design requirements and implementation considerations for future deployment. Our work focused on *early* prototyping to develop technology solutions and build a shared understanding of the social connection needs in this population. Our long-term goal is to develop smart speaker–based applications called *Voice2Connect* for deployment in affordable housing to enhance social connectedness among their residents.

## Methods

### Study Design

We conducted a UCD study to engage residents in developing smart speaker–based applications for social connectedness. Our process consisted of three major stages conducted between April 2021 and April 2022: (1) needs assessment and idea generation, (2) iterative prototype development, and (3) validation and implementation planning (Table 1).

**Table 1.** Study design overview.

Stage and phases	Activities	Participants	Key outcomes
Stage 1: Needs assessment			
Phase 1	Focus group discussions Video introduction to smart speakers 6-8-5 brainstorming exercise Pictorial sheet ideation	25 older adults (5 groups; 12 users and 13 nonusers)	Social connection needs 153 design ideas User requirements
Stage 2: Iterative development			
Phase 2	Team synthesis of phase 1 data Design Strategies Map creation Initial scenario development	Research team	4 functional categories 7 initial scenarios
Phase 3	Scenario presentations via role-play Feedback focus groups	18 older adults (5 focus groups; 8 users and 10 nonusers)	Design preferences Concerns identified Improvement suggestions
Phase 4	Matrix analysis of feedback Scenario refinement	Research team	7 refined scenarios
Stage 3: Validation			
Phase 5	Refined scenario presentations Validation focus groups Implementation planning	17 older adults (4 groups; 5 users and 12 nonusers)	Validated prototypes Deployment recommendations

### Setting and Participants

We recruited participants from 3 housing buildings in Richmond, Virginia, United States (referred to as housing A, B, and C) that serve older adults with limited income and individuals with disabilities. Our research team had established relationships with these communities through prior collaborative research and wellness programs. Eligible participants were older than 55 years, lived alone, could speak and understand English, and were able to participate in all study activities. We excluded individuals with severe sensory impairments preventing engagement with audiovisual materials, terminal illness, or a score ≤8 on a short version of the Montreal Cognitive Assessment indicating moderate to severe cognitive impairment. Participants were divided into 2 groups based on smart speaker experience: users (n=13) who had been using Amazon Echo Dot devices deployed by housing A since January 2020, and nonusers (n=16) from all 3 housing sites.

We recruited participants through multiple channels including housing management referrals, community wellness program participant lists, on-site information sessions, and flyers. Due to pandemic-related visitor restrictions, eligibility screening was conducted by phone, during which we also collected demographic and technology experience information. Detailed descriptions of recruitment procedures can be found elsewhere [30].

### Ethical Considerations

The Institutional Review Board of Virginia Commonwealth University determined that this project posed minimal risk and waived written informed consent (HM20020008).

### Procedures

We conducted focus groups with older adults in phases 1, 3, and 5 to perform an iterative co-design process and gather data. The groups were stratified by housing building and current smart speaker use (Table 2).

**Table 2.** Focus group stratification by phase and participant group (older adult residents).

Phase and building	Smart speaker user	Values, n	Total values
1			25
A	Yes	6	
A	Yes	5	
B^a^	No	6	
A	No	5	
C	No	3	
3			18
A	Yes	4	
A	Yes	4	
A	No	4	
B	No	4	
C	No	2	
5			17
A	Yes	5	
A	No	2	
B	No	6	
C	No	4	

a One nonuser resident from building A attended this meeting.

#### Stage 1: Needs Assessment and Idea Generation (Phase 1)

We conducted 5 focus groups (n=25) between April and July 2021. Due to pandemic restrictions, we held the first 3 meetings in a campus lecture room, providing van transportation since most participants lacked reliable transportation. Safety protocols included temperature checks and mask requirements.

Each 1‐ to 1.5-hour session consisted of 2 parts. First, we explored participants’ needs for establishing and maintaining meaningful social contacts and their desired outcomes from smart speakers. To help nonusers understand the technology, we presented a 5-minute video demonstrating basic smart speaker tasks (weather checking, drop-in calls, and dictionary functions). Second, we facilitated rapid brainstorming using the 6-8-5 design method [31], where participants generated 6‐8 ideas in 5 minutes. Participants worked in mini groups of 2‐3 with a facilitator, sharing ideas and asking clarifying questions. We used pictorial sheets (Multimedia Appendix 1) where participants articulated specific social connectedness tasks they wanted to accomplish using smart speakers. After 2 brainstorming rounds, mini groups presented key ideas to the larger group. Audio-recorded sessions were transcribed verbatim and analyzed as described in the Data Analysis section.

#### Stage 2: Iterative Prototype Development (Phases 2-4)

##### Phase 2: Initial Scenario Development

The research team synthesized phase 1 data through multiple team meetings, creating a Design Strategies Map that linked user needs with potential smart speaker–based solutions. Through triangulation of focus group data and pictorial sheet ideas, we identified 4 main functional categories: checking-in, social companion, community involvement, and wellness check. We developed 1‐2 scenarios within each category—short stories depicting user characteristics, context of use, and specific technology-based tasks to achieve goals [32,33]. Scenarios incorporated voice interactions with “Alexa” and were written at a third-grade reading level to ensure readability and accessibility.

##### Phase 3: Evaluation Focus Groups

We conducted 5 focus groups (n=18) in October 2021, presenting initial scenarios through role-play demonstrations by 2 team members. After each scenario, we solicited feedback on design perceptions, willingness to use, improvement suggestions, and concerns.

##### Phase 4: Scenario Refinement

Phase 3 focus group recordings were transcribed verbatim and analyzed as described in the Data Analysis section. We identified key suggestions for each scenario and revised prototypes accordingly.

### Stage 3: Validation and Implementation Planning (Phase 5)

We conducted 4 focus groups (n=17) in April 2022 to validate refined scenarios. Team members again used role-play to present scenarios, explicitly noting incorporation of previous feedback. We solicited perceptions, desired additional features, and implementation recommendations. Sessions were transcribed verbatim and analyzed as described in the Data Analysis section. Throughout the study, participants received US $20 gift cards at each session. Focus groups were limited to 6 participants to encourage initiated conversations while minimizing groupthink [34].

### Measures

Before phase 1, we conducted a phone-based survey to collect data on demographics, technology experience, social well-being (social isolation, loneliness, and satisfaction with current social interaction levels; Multimedia Appendix 2), and other aspects of health such as functional status, depression, sleep, and physical activity. This manuscript reports only on demographics, technology experience, and social well-being.

### Data Analysis

We used a combination of inductive and deductive qualitative approaches across the 3 stages of the UCD process, selected to match the analytic goals of each phase (Table 3).

**Table 3.** Focus group data analytic approach.

Stage	Phase	Analytic approach
Stage 1	Phase 1	Inductive thematic analysis; content analysis of pictorial sheets
Stage 2	Phases 3‐4	Deductive content analysis; matrix display
Stage 3	Phase 5	Deductive content analysis

#### Phase 1 (Needs Assessment)

Three team members conducted inductive thematic analysis [35] of focus group transcripts to explore participants’ social connection needs and identify user preferences for low-fidelity prototype development. Transcripts were imported into Atlas.ti (Lumivero, LLC) software for coding. They independently reviewed transcripts multiple times and met to discuss how participants wanted to use smart speakers for social connectedness and general well-being. These preliminary ideas became codes (eg, how to stay in contact, feeling lonely or isolated, and user needs and preferences), forming the basis for an initial codebook that included code definitions. The team had regular meetings to discuss coding and listed all design ideas mentioned by participants and relevant quotes. We used Miro for affinity mapping to cluster ideas into emergent categories. Rigor strategies included collaborative coding with consensus discussion, peer debriefing with external stakeholders, an audit trail of analytic decisions, and rich description with illustrative quotes. Detailed user challenges with voice user interface on smart speakers and social isolation and loneliness experiences among these participants are published elsewhere [36].

We conducted a content analysis [37,38] of pictorial sheet data using a deductively developed coding framework of 8 categories: daily assistance, health and safety, socializing with others, learning, companionship with a voice agent, entertainment, religion, and miscellaneous. One researcher (NM) assigned category labels to all ideas, which were subsequently reviewed and validated by another researcher (JC). They reconciled any discrepancies through a team discussion, after which the total number of smart speaker–based tasks for each category was calculated.

#### Phases 3‐4 (Prototype Evaluation and Refinement)

Two researchers (JC and NM) independently conducted deductive content analysis [37] of phase 3 focus group transcripts using the 4 functional categories established in phase 2 as an organizing framework. A matrix display approach [39] was used to systematically arrange findings by domain, enabling efficient comparison of design preferences and recommendations across focus groups [40]. They (JC and NM) read transcripts multiple times and made notes independently in a pre-agreed template. Then they compared their coding results and reconciled any discrepancies. Based on this discussion, they identified key suggestions for each scenario, presented these to the whole team to get feedback, and then made refinements to the prototype scenarios.

#### Phase 5 (Validation)

Two team members analyzed transcripts following the phase 4 approach, using deductive content analysis to identify themes relevant to system deployment and implementation planning.

## Results

### Participant Characteristics

Twenty-nine older adults participated in 1 or more focus group sessions (Table 4). The sample was predominantly African American (n=23, 79%), with 55% (n=16) male. Out of 29 participants, 20 (69%) participants had completed high school education or less. While 21 (72%) participants owned smartphones, only 15 (52%) participants had computer or laptop access, and 12 (41%) participants had never used the internet. Thirteen (45%) participants were smart speaker users at study inception, 11 (85%) of whom reported prior internet use and 2 (15.4%) did not. Among the 11 participants with prior internet use, the majority (n=8, 73%) were daily internet users.

**Table 4.** Participant characteristics (N=29*)*.

Characteristics	Participants
Age (years), mean (SD); range	70 (6.8); 60‐87
60‐69	12 (41.4)
70‐79	14 (48.3)
≥80	3 (10.3)
Sex, n (%)	
Female	13 (44.8)
Male	16 (55.2)
Race, n (%)	
African American	23 (79.3)
White/Caucasian	5 (17.2)
Other	1 (3.4)
Hispanic/Latino, no	29 (100)
Education, n (%)	
Less than high school	7 (24.1)
High school diploma/general education development	13 (44.8)
Some college	6 (20.7)
Bachelor’s degree	1 (3.4)
Graduate or professional degree	2 (6.8)
Smart speaker ownership, yes, n (%)	13 (44.8)
Smartphone ownership, yes, n (%)	21 (72.4)
Laptop/computer ownership, yes, n (%)	15 (51.7)
Ever used internet, n (%)	
Yes	17 (58.6)
Never	12 (41.4)
Daily internet users, n (%)	13 (44.8)
Social isolation measured by LSNS-6^a^, mean (SD); range	15 (SD 6.7); 3‐24
Social isolation (LSNS-6 <12), yes, n (%)	8 (27.6)
Loneliness measured by UCLA^b^ Loneliness Scale Short Form, mean (SD); range	4.6 (SD 1.9); 3‐9
Loneliness (UCLA-3 ≥ 6), yes, n (%)	9 (31.0)
Participation in any organizations, religious groups, or committees; yes, n (%)	12 (41.4)
Satisfaction with the current social relationships, n (%)	
Very dissatisfied	10 (34.4)
Somewhat dissatisfied	3 (10.3)
Satisfied	16 (55.2)

a LSNS-6: Lubben Social Network Scale-6 items.

b UCLA: University of California, Los Angeles.

At baseline, participants had a mean Lubben Social Network Scale-6 score of 15 (SD 6.7), with 8 (28%) out of 29 participants meeting the threshold for social isolation (Lubben Social Network Scale-6 score of <12). Mean University of California, Los Angeles (UCLA) Loneliness Scale-Short Form score was 4.6 (SD 1.9), with 9 (31%) out of 29 participants meeting the threshold for loneliness (UCLA Loneliness Scale-3 ≥6). While just over half of the participants (16/29, 55%) reported being satisfied with their current social relationships, 34% (10/29) reported being very dissatisfied and 10% (3/29) somewhat dissatisfied, and fewer than half (12/29, 41%) reported participating in any organizations, religious groups, or committees.

### Stage 1: Understanding Social Connection Needs and Generating Ideas

#### Social Connection Context and Needs

Focus group discussions revealed multiple factors driving the need for enhanced social connections among residents. Participants described how the pandemic disrupted existing social ties, leaving many feeling isolated. They expressed willingness to help fellow residents experiencing isolation but lacked mechanisms to do so. Many had restricted social networks in general due to limited transportation, mobility constraints, and inadequate access to information about community activities. A critical finding was that participants’ social connection needs were inseparable from safety and survival concerns related to living alone. As one participant explained:

*That would be the best thing we need for Alexa to do. To let everybody—where everybody can say I’m okay, or they check in. Somebody from the office or somebody calls and checks on everybody*.

[Group 1, smart speaker user]

Participants emphasized that unexpected health events were a constant worry, creating a need for check-in systems that went beyond traditional socialization support.

#### Brainstormed Design Ideas

Twenty-four participants generated 153 ideas on pictorial sheets (MultimediaAppendices 3  4), with individual contributions ranging from 1 to 16 ideas. The mean number of ideas developed by smart speaker users and nonusers was similar (users: mean 6.6, SD 3.8; nonusers: mean 6.2, SD 5.2). The most frequent categories were Daily Assistance (n=41, 26.8%) and Health and Safety (n=34, 22.2%). Social connectedness generated 35 total ideas across 2 categories: Socializing With Others (n=26, 17.0%) and Companionship With Virtual Agent Alexa (n=9, 5.9%). Additional categories included Learning (n=28, 18.3%), Entertainment (n=7, 4.6%), Religion (n=3, 2.0%), and Miscellaneous (n=5, 3.3%) purposes. Examples of social connection ideas included: “Talk to other residents” and “Call my daughter,” “Alexa could call my friends and tell my friends I said hi, and tell them to call me,” “Help me check on my health...If I’m feeling a certain way, tell me what you think is wrong,” and “What if there was a way to send out a message saying, ‘I’m going to go walk. Anyone want to go walk with me?’”

### Stage 2: Co-Designing Smart Speaker–Based Applications for Social Connectedness

#### From User Needs to Design Principles

Through synthesis of focus group transcripts and pictorial sheet data, we developed a Design Strategies Map (Table 5). This analysis revealed overarching design principles: (1) the system should support both social connections and safety while living independently, (2) the system should help build stronger community cohesion, and (3) the design should recognize differing needs across individuals and housing contexts. These principles guided development of 4 functional categories with specific use cases designed to address the social-living context of affordable housing residents aging alone (Multimedia Appendix 5).

**Table 5.** Design Strategies Map linking older adults’ needs to smart speaker–based application^a^.

User need theme	Illustrative quotes	Design response
Safety and Emergency Support While Living Alone	"That would be the best thing we need for Alexa to do...where everybody can say I’m okay, or they check in. Somebody from the office or somebody calls and checks on everybody.” [User, Group 1] “If we had Alexa, (we could say) ‘Alexa, I feel like I’m gonna fall out. Send 911.’ A lot of people here have nobody they can talk to.” [Nonuser, Group 5]	Checking-in function Peer-to-peer wellness checks Management check-in programs Emergency contact system Reminders to connect with others
Companionship and Emotional Support	“It’d be nice if I could have something to talk back to me.” [User, Group 2] ‪“Sometimes I felt down, and I talked to Alexa, and she gave me some good answers...‪when I’m lonely, that’s for sure.” (Nonuser, Group 3) “It could help you—if you’re a person with depression and anxiety, you could talk to Alexa to have some company. That would help you have company—have somebody to talk to. Especially if you don’t come out and get no fresh air or walk nowhere, where you take walks and see people and speak to them.” [Nonuser, Group 4) “You would need somebody to talk to. Can Alexa read books?” [Nonuser, Group 5]	Social companion function Conversational AI^b^ for daily interaction Positive affirmations and greetings Conversational AI reading books aloud to user and encouraging discussions Recall of past conversations
Community Connection and Social Participation	“What if there was a way to send out a message saying, ‘I’m going to go walk. Anyone want to go walk with me?...You might decide to go, right?” [User, Group 1] “Alexa could call my friends and tell my friends I said hi, and tell them to call me.” [Nonuser, Group 4] “Keeps me in touch with my daughter.” [Nonuser, Group 5] “That would be helpful to me if it will remind me to call my mom.” [Nonuser, Group 5] “I don’t want to just think this [building] is where I’m gonna die at...[We] want something lively.” [Nonuser, Group 5]	Community involvement function Virtual bulletin board for events Coordination of group activities Information about local resources Facilitation of meetups and outings
Health Monitoring and Wellness Support	“Help me check on my health, such as my temperature, blood pressure....If I’m feeling a certain way, tell me what you think is wrong.” [Nonuser, Group 3]	Wellness check function Integration with wearables Activity pattern monitoring Mood and loneliness detection Prompts for intervention

a These ideas were identified through phase 1 focus groups and brainstorming activities. Design responses represent the 4 functional categories developed for prototype scenarios.

b AI: artificial intelligence.

#### Four Functional Categories of Smart Speaker–Based Applications

##### Category 1: Checking-In (2 Scenarios)

Participants identified checking-in as the most important feature. This included both peer-to-peer and management-to-resident check-ins to address living-alone concerns and emergency support needs, focusing on confirming that a resident is present and safe. As one participant noted: “A lot of people here have nobody they can talk to” [Group 5, nonuser]. The scenarios enabled residents to connect with neighbors through voice commands and established opt-in check-in programs with housing management.

##### Category 2: Social Companion (2 Scenarios)

Conversational AI could offer companionship, emotional support, and continuity by recalling past interactions. Participants recognized that this could help residents feel less lonely: “Sometimes I felt down, and I talked to Alexa, and she gave me some good answers...when I’m lonely, that’s for sure” [Group 3, nonuser]. Scenarios depicted personalized interactions where Alexa engaged in book discussions and daily conversations with residents.

##### Category 3: Community Involvement (2 Scenarios)

The technology could facilitate participation by sharing local information (eg, bus routes and community events), enabling virtual gatherings, or arranging meetups. Participants wanted to build “something lively” rather than viewing their housing as purely a place to age in isolation. Scenarios included virtual bulletin boards for community activities and coordinated group trips to essential destinations. Housing providers’ role was assumed to coordinate and manage these programs while Alexa’s role was access facilitation.

##### Category 4: Wellness Check (1 Scenario)

Integration with activity tracking devices such as fitness bands capable of monitoring movement, sleep, and inactivity could enable system-initiated health monitoring that detects changes in activity levels as potential signals of physical and emotional health status, prompting the user toward individual action or connection with others (eg, health care providers and significant others). This scenario assumed that participants would have access to such devices, which would communicate activity data to the smart speaker. We developed 7 initial scenarios across these categories based on iterative refinements based on team review (Multimedia Appendix 6), written at accessible reading levels and featuring familiar names to enhance relatability.

### Refining Prototypes Through User Feedback

Phase 3 evaluation revealed both enthusiasm and concerns. Participants appreciated applications that could foster connection and help them stay informed about housing events. They strongly preferred technology facilitating human connection over purely automated interactions. The social companion features were seen as useful for those experiencing loneliness, although some worried about replacing rather than supplementing human contact.

Privacy and obtrusiveness emerged as primary concerns. Participants worried about other residents or management checking-in at any time, unintentional sharing of location and activity information, and technology exerting excessive control (described as “too Orwellian” by one group). Responses to the wellness check scenario indicated that participants were receptive to the use of wearable-based data for monitoring physical inactivity and detecting potential falls. However, they expressed a clear preference for systems that prompt rather than diagnose, flagging activity changes and facilitating human connection, rather than inferring emotional states from sensor data.

Participants provided concrete improvement suggestions for each scenario (Multimedia Appendix 7). In summary, these include (1) user control—add “do-not-disturb” features and allow selection of check-in frequencies, (2) privacy protection—create close contact lists rather than building-wide access and ensure that health information is not shared during check-ins, (3) natural interaction—make voice interfaces sound conversational rather than mechanical, (4) minimal management oversight—limit housing management role to essential check-ins only, and (5) personalization options—allow users to set speaking times, choose conversation perspectives, and toggle features on/off.

The depth and quality of participant contributions did not differ by prior smart speaker experience. Analysis of focus group interviews across all 7 design scenarios revealed comparable levels of engagement between smart speaker users and nonusers. Per focus group, both groups generated similar numbers of positive themes (users: 11; nonusers: 11) and design recommendations (users: 14; nonusers: 11.3). The major design themes, including privacy and user control, perceived usefulness for socially isolated residents, trusted contact network preferences, and voice-based security, emerged consistently across both groups. While users occasionally drew on their lived device experience, nonusers contributed equally substantive and actionable recommendations, including novel suggestions such as video interfaces for hearing-impaired residents, information integration, and peer activity coordination via Alexa.

**Figure 1.**
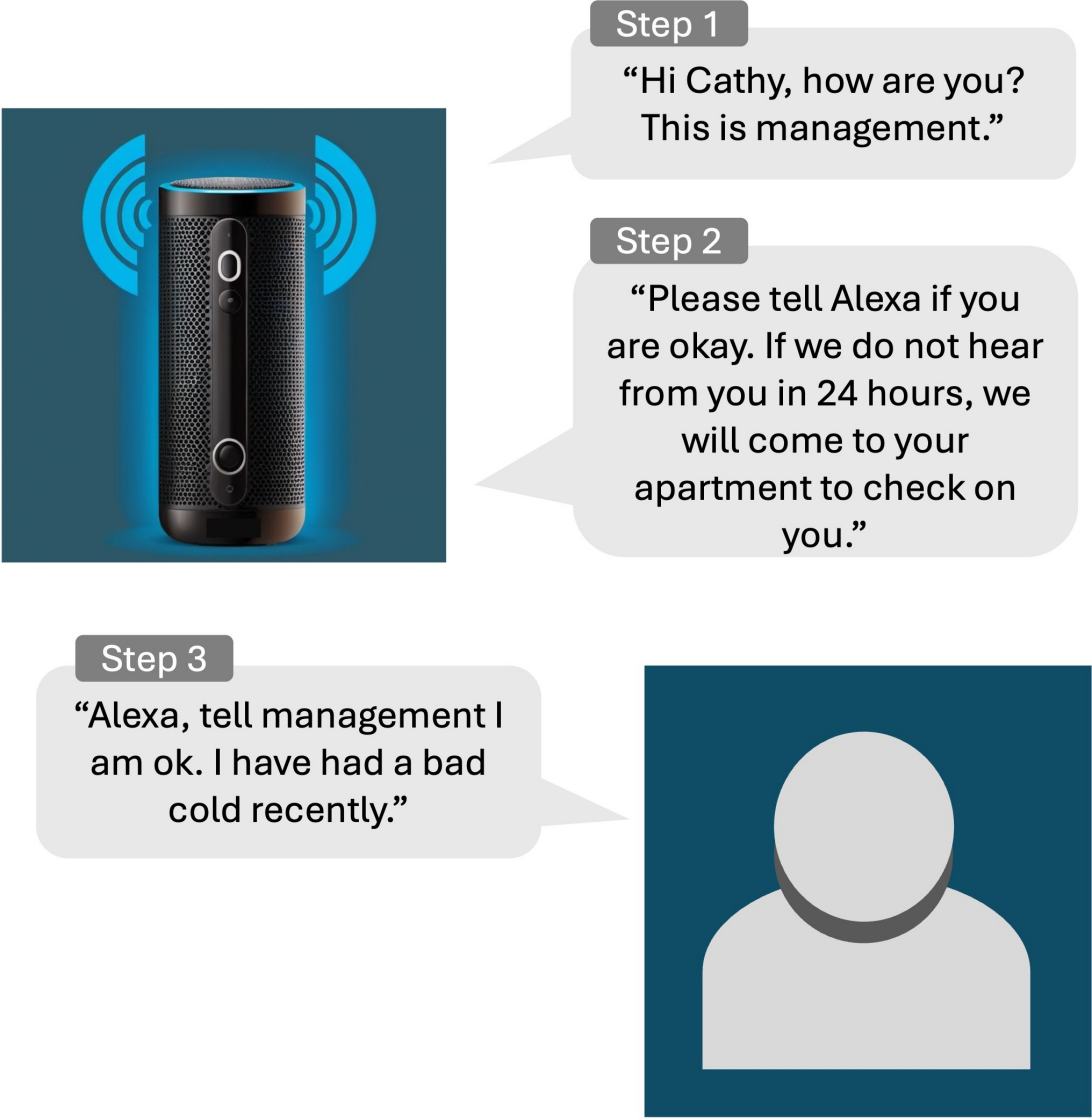
A graphic representation of a Voice2Connect prototype in the checking-in category. This figure shows verbal interactions between user and voice agent.

Based on this feedback, we revised all 7 scenarios (Textbox 1). Key changes included adding opt-in or opt-out capabilities, do-not-disturb functions, contact list controls, and explicit privacy protections. For example, in the management check-in scenario, we specified that Alexa would confirm resident status without sharing additional details the resident mentioned (eg, having a cold). Figure 1 shows a graphic representation of a scenario in the checking-in category.

Textbox 1. **Refined scenarios of use for the Voice2Connect system prototypes. Italicized text shows the changes made based on the feedback from phase 3 focus groups.**

**Category 1: Checking-in**

***Scenario 1: Using Alexa to talk to your neighbor or friend***

Tom, 78-year-old man, uses the smart speaker to check in on others in the building. Tom has not heard from or seen his neighbor Jeanne in 2 weeks, but he knows that Jeanne also has an Alexa Dot, and *he and Jeanne have added one another to their contact lists*. So he speaks to Alexa to see whether Jeanne is doing well: “Alexa, connect me to Jeanne” and then he is connected to Jeanne. Tom asks Jeanne, “Hey, how are you doing? I just want to make sure you are well.” Tom and Jeanne chat briefly through the speaker to share what is going on with them. Two days later, Tom is watching TV and *he doesn’t want to get bothered, so he turns on the Do Not Disturb Feature. When Jeanne tries to contact him, there is an auto-message from Alexa:* “Tom has turned on ‘Do Not Disturb.’”

***Scenario 2: Check-ins from the management***

*The building management started a resident check-in program through Alexa. Cathy opted into the wellness check-in service, and she chose management daily check-ins. Tom also opted into the check-in service and he chose weekly check-ins*. The office manager put an announcement on the Alexa of check-in program participants in the building: “Hi Cathy, how are you? This is management. Please tell Alexa if you are ok. If we do not hear from you in 24 hours, we will come to your apartment to check on you.” Cathy said, “Alexa, tell management I am ok. I have had a bad cold recently.” Alexa informs the management that Cathy is okay and *doesn’t say anything about Cathy’s bad cold*.

**Category 2: Social companion**

***Scenario 1: Being a friend with Alexa***

George is a widowed 85-year-old man and lives alone. *He wants to have an individualized interaction with Alexa, so he chooses the highest level of personalization*. George’s children come and visit him on the weekends, but during the week he has little social interaction and often feels lonely. Alexa detects when George gets out of bed and greets him by saying “Good Morning, George, how did you sleep?” George replies saying, “I slept well. Alexa, can you read me the next chapter of my Steven King book at 10 am?” At 10 AM, Alexa says, “Here is where we left off reading yesterday” and continues to read the book aloud to George. After some time, George says, “Alexa, stop reading. This book is getting very good.” Alexa replies, “It is. What do you think is going to happen next?” George replies, and Alexa says, “That is possible, we should read again tomorrow.”

***Scenario 2: Conversation between you and Alexa***

John has arthritis in his knees and hips, making walking and standing for long amounts of time painful for him, so he likes to stay at home most of the time. Getting out in the community and connecting with other people has been difficult because of John’s limited mobility. John likes to talk to Alexa. In the mornings he will say “Good morning, Alexa,” and Alexa will reply, “Good morning, John. Today is going to be a blessed day.” John is able to reply, “Every day is a blessed day.”

John likes to talk out loud about what is happening in his favorite television show and movies. Alexa remembers what John says is happening in the movie and is able to talk with him about it. “John, that is a popular movie. Online it has hundreds of 5-star reviews. One person left a review and said ‘this is one of my favorite movies. I was not a big fan of the ending, but overall the plot was good,’” “Alexa, I agree, but it was still a good movie.”

**Category 3: Social participation**

***Scenario 1: Getting involved in the community***

Alice, a 75-year-old woman, recently moved into a new apartment building to be closer to her children. *Alice wants to make new friends in the building, so she has opted into the community events bulletin board. When Alice comes home from the grocery store, she sees a light on her Alexa, telling her there is an announcement. Alice asks Alexa,* “Alexa, what is the announcement?” *Alexa says,* “There is a Spanish class today at 3 pm. Would you like to hear other activities happening today?*” Alice says,* “yes, please,” *and Alexa reads Alice the list of community activities. Alice says,* “Alexa I think I want to try that Spanish class. Remind me when it is time.” *Later at 2:50 PM, Alexa says,* “Don’t forget, Spanish class starts in 10 minutes.” On Sunday, Alexa says to Alice “Good morning Alice, there is the weekly church service going on in the community room downstairs. Everyone in the building is invited.”

***Scenario 2: Meetups***

David is a new resident of affordable senior housing and recently got an Alexa. After settling into his new apartment, *he opted in to a virtual bulletin board to see what events are happening in the building. David asks Alexa, “*Is there any activity going on today?*”* Alexa says, “John and Martha will walk to a park at 3 pm today for exercise. Do you want to join?” David says, “Yes, put me on the list.” *Alexa reminds David 30 minutes before, “*David, you will need to meet them at the entrance at 3 pm.”

A couple of days later, David made a list of grocery items he needed. Being new to Richmond, David did not feel comfortable going alone. He said, “Alexa, can you ask if anyone needs to go to Kroger today? We can all take the bus together. *Also, add this to the virtual bulletin board so other people know about this*.” Alexa replies, “Martha will go to Kroger with you today at 2 pm. You can take the #3 bus at 1:30. It will pick you up in front of the building.”

**Category 4: Wellness check and mood detection**

***Scenario 1: Mood detection***

Paula, a 69-year-old woman, has been living in her apartment building for 4 years. She would describe herself as fairly active and social with a few good friends living in the same apartment building. Recently, her only child and grandchildren moved further away from Paula, making it more difficult to see each other. *Paula is worried about becoming lonely, so she opts into the loneliness detection feature*. A few weeks later, Paula is seeing her friends less frequently, sleeping in later, taking naps, and is spending more time watching TV. Alexa says “Paula, the data from the Fitbit tell that you have not been as active as you normally are. How are you doing? Would you like me to connect you with one of your friends or your doctor?” *Paula says,* “Well, Alexa, I don’t think I need the doctor, but I haven’t been very active recently and am feeling a little lonely.” Three days later, Paula still has not left her apartment. *Alexa says,* “Paula, you still seem to be less active than usual. Would you like me to connect to your friend or read the list of activities on the bulletin board?” *Paula agrees, and Alexa connects Paula to her friend to make plans for a walk*.

### Stage 3: Validating Final Prototypes and Planning Implementation

#### Overview

Participants expressed varied but generally positive opinions about refined scenarios. Overall, they appreciated the vision of using smart speakers to foster a more connected and livable community, although enthusiasm varied by scenario. Features requiring movement or mood detection to identify emotional health risks were viewed most skeptically, with concerns about excessive technological control over daily life.

#### Crosscutting Themes From Validation Sessions

##### Personalization as Essential

Participants emphasized the importance of controlling features rather than having a one-size-fits-all system. Suggestions included setting specific times for Alexa to speak, choosing conversation styles or perspectives (eg, “Christian perspective”), and selecting which physiological metrics to monitor.

##### Safety Remains Paramount

Across nearly all scenarios, participants referenced potential health emergencies, particularly for residents in poorer health or facing adverse outcomes. The checking-in and wellness check functions were especially valued. As one participant noted, “That’s better than somebody knocking on your door to see if you’re okay” [Group 4].

##### Preventing Replacement of Human Connection

Participants stressed the need for safeguards against AI technology replacing human interaction or enabling abusive behaviors. They wanted technology that facilitated human connection rather than substituted for it.

## Discussion

### Principal Findings

This study used an iterative UCD process to engage older adult residents in affordable housing in designing prototype scenarios for smart speaker–based applications to promote social connectedness. Throughout 3 stages of co-design, we developed 7 scenarios across four functional categories: (1) checking-in for safety verification, (2) conversational AI as social companions, (3) community involvement, and (4) wellness monitoring. These scenarios provide a foundation for developing a technology platform tailored to address the specific social isolation and loneliness experienced by underserved older adult populations, who often face distinct challenges in locating and accessing the social and financial resources necessary for aging in place. Baseline social well-being data underscore this need. While more than half reported satisfaction with their current social relationships, a meaningful subgroup was experiencing clinically significant loneliness and social isolation, lending ecological validity to their strong interest in features supporting social connectedness in their living communities.

The findings of this study can be interpreted through the lens of Self-Determination Theory [41], which posits that human motivation and well-being are grounded in 3 psychological needs: autonomy, competence, and relatedness. Self-Determination Theory maps meaningfully onto the design preferences and concerns expressed by participants across all phases. The demand for customization, opt-in controls, do-not-disturb features, and close contact lists reflects a fundamental need for *autonomy*, the desire to maintain personal agency over one’s social environment and information sharing. Participants’ requests for interfaces that are simple, conversational, and responsive to their voice reflect the need for *competence*, the ability to engage effectively with technology without feeling overwhelmed or excluded. Finally, participants’ desire for building a livable community for themselves and peers and for preserving human relationships in the loop reflects the need for *relatedness*, belongingness, and meaningful connection with others. Designing smart speaker–based applications that satisfy these needs may be essential not only for usability but for sustaining intrinsic motivation for continued technology engagement and well-being outcomes in this population.

### Social Connectedness Inseparable From Safety and Survival

Throughout the design process, it became clear that the study’s focus on social connectedness strongly resonated with participants due in part to the isolation they experienced during the COVID-19 pandemic, a period during which residents faced significant restrictions on in-person social interaction. The strong participant interest in peer check-ins and community engagement features may partly reflect this context, and it is plausible that the relative prioritization of these features could differ in a postpandemic sample. Importantly, however, a critical insight emerged: for affordable housing residents living alone, social connection needs cannot be separated from safety and survival concerns. Participants emphasized that any technology system must address both relationship-building and emergency support, reflecting the reality that all residents live alone and some face immediate safety vulnerabilities, including declining health and fall risk, that may arise at times when human assistance is not immediately available.

This dual emphasis shaped our design approach fundamentally. Rather than creating purely social applications, we prioritized checking-in functionality that serves both social and safety purposes. This finding aligns with research suggesting that older adults’ technology adoption is strongly influenced by whether innovations address their most pressing daily concerns [42]. This study shows that for affordable housing residents with limited support networks, safety vulnerabilities are not abstract future concerns but immediate realities shaping their daily experiences of living alone [43].

### Building Social Infrastructure in Affordable Housing

Our findings indicate that residents were genuinely interested in making a livable community within their housing by building social infrastructure [44], not just to address social needs but also to promote safe, independent living. Participants suggested various ideas, including virtually connecting with individuals in need, conducting remote safety check-ins, advertising community events, and facilitating coordinated trips to essential destinations (eg, grocery stores). Participants were interested in expanding and enhancing social networks within and beyond their housing community. Research suggests that digital tools connecting older adults to existing social networks may not significantly increase social activity for individuals lacking networks and at risk of social isolation [45]. However, tools designed to facilitate new social connections or broaden access to community resources can play a critical role in addressing these gaps [46]. Our scenarios address this through community-building features (eg, virtual bulletin boards, coordinated meetups, and shared activity planning) that help residents discover and connect with neighbors who share similar needs and interests.

The emphasis on community involvement also reflects participants’ recognition that their housing buildings could become more than just places to live in isolation. They wanted technology that would help them build “something lively,” a community with regular interaction, mutual support, and shared activities. This vision extends beyond typical conceptualizations of smart speakers as individual assistive devices and positions them as one component of a broader, community support ecosystem that can facilitate communication about opportunities and access to them, while the core work of building and sustaining community remains a fundamentally human endeavor. Importantly, technology alone is insufficient to establish such infrastructure. The foundation must be built through human-centered programming, dedicated staffing, and sustained relational investment. Smart speaker–based applications, therefore, should be understood not as substitutes for this work but as complementary tools that facilitate awareness of and access to existing and emerging community resources, functioning as bridges to human connection.

### Engaging Underserved Communities in Technology Design

To our knowledge, this is one of few studies using UCD with older adults of lower socioeconomic status. Such populations are often excluded from design and testing processes for new technologies conceptualized to improve quality of life and health [47], probably due to concerns about their ability to engage meaningfully and provide valuable input. Limited access to ICT and low digital literacy also may have contributed to the underrepresentation of these communities in technology research and design initiatives. The limited participation of marginalized communities perpetuates the exclusion of their perspectives [48] and contributes to disparities in digital health access and outcomes. Given that widespread digital technology access has created new opportunities for self-management, quality of life, and social well-being in older adults [49-51], recognizing the importance of engaging community members of underrepresented populations in a technology development and testing process is essential.

Our study demonstrates that older adults in underserved communities, including those with limited prior technology experience, can actively participate in and meaningfully contribute to technology design processes. Our participants across age groups, technology experience levels, and housing communities demonstrated enthusiasm and active engagement and expressed a sense of ownership in the design sessions, contributing valuable insights that shaped prototype development. The 153 ideas generated during brainstorming activities, the nuanced feedback on privacy and control features, and the concrete implementation recommendations all demonstrate that concerns about this population’s ability to engage in design work are unfounded.

### Methodological Contributions: Adapting UCD for Underserved Communities

Our research has methodological implications for UCD research with and for underserved communities. The 3-stage iterative approach we used successfully balanced rigorous design principles with the unique contexts and abilities of participants. Our inclusive methods included rapid brainstorming sessions, facilitated mini-group discussions to ensure that everyone had a voice and that discussions remained focused and productive, audiovisual materials to enhance nonusers’ understanding of the target technology and stimulate idea generation, scenario creation and role-play demonstrations to reduce uncertainty about the technology and facilitate discussions around privacy and security, and iterative design revision informed by continuous feedback.

These methods were thoughtfully chosen based on the literature [31-33] and the team’s extensive experience working with affordable housing communities. Beyond specific UCD techniques, the team prioritized creating a comfortable, nonjudgmental environment where participants, especially those not familiar with smart speakers, could freely propose ideas, suggest changes to scenarios, and identify concerning features. We found that using pictorial sheets allowed participants with varying literacy levels to express ideas, while mini-group formats prevented more vocal participants from dominating discussions.

This study also demonstrates the need for flexibility in balancing rigorous design principles with the unique contexts and abilities of participants, as previous research demonstrated [32]. For example, during phase 1, we opted not to conduct mini-group sessions with focus group 5 (nonusers) after observing their difficulty engaging in technology-related discussions. Instead, we adapted our approach to facilitate a large group discussion focused on preferred functionalities for social connectedness, emphasizing their role as co-designers. This adaptive method adds important considerations specific to a UCD process with underserved populations, suggesting that methodological fidelity should sometimes yield to participant comfort and authentic engagement.

The established trust relationships our research team had developed through prior community-engaged work [52] proved essential to recruitment and retention. This suggests that successful UCD with marginalized communities requires sustained institutional commitment beyond individual research projects, and researchers cannot parachute in for design sessions without prior relationship-building and expect meaningful engagement.

### Privacy, Control, and Trust as Essential Design Requirements

Participants’ privacy concerns emerged consistently across all 3 stages and shaped significant design revisions. Their worries about unintentional information sharing, management surveillance, and loss of autonomy led to specific requirements: opt-in or opt-out controls, do-not-disturb features, close contact lists rather than building-wide access, and explicit privacy protections ensuring that sensitive information would not be shared with management or other residents. These concerns reflect broader issues of power, trust, and control that are particularly salient for minority, low-income older adults who may have experienced systemic marginalization and surveillance [53]. The description of mood detection features as “too Orwellian” signals deep discomfort with technologies that could exert control over their lives without consent. Our design iterations directly addressed these concerns by centering user control and transparency.

Importantly, participants distinguished between features they found helpful (checking-in on neighbors and being reminded to connect with others) and those they found intrusive (automatic mood detection and unrestricted management access). This nuanced feedback demonstrates why participatory design is essential because researchers and technologists might assume that more automated, comprehensive monitoring would be beneficial, while users clearly articulated the need for boundaries and personal agency.

### Balancing Human Connection and Technological Companionship

Participants expressed interest in voice agent companionship while simultaneously voicing concerns about replacing human interaction. This tension reflects broader societal questions about the role of AI in addressing loneliness and social isolation. Participants recognized that for those experiencing severe isolation, having “somebody to talk to” through Alexa might be “better than nothing,” while also insisting on the primacy of human relationships.

Our scenarios attempted to navigate this tension by positioning voice agents as facilitators of human connection rather than replacements for it. For example, the social companion scenarios included Alexa suggesting the user connect with friends for walks rather than only offering its own companionship. The wellness check scenario prompted connection to friends or health care providers rather than only providing automated support. This design approach aligns with emerging ethical frameworks for AI companionship that emphasize augmentation rather than substitution [18]. However, participants’ concerns suggest that implementation will require careful monitoring to prevent vulnerable, isolated individuals from becoming overly reliant on AI interaction at the expense of human relationships. Future development should include safeguards that encourage and facilitate human connection rather than allowing AI to become the primary or sole social contact.

### Implementation Considerations and Future Directions

The varying levels of enthusiasm across scenarios suggest that a modular, customizable system would be most appropriate. Rather than implementing all 7 scenarios uniformly, housing communities should be able to select and configure features based on resident preferences, building infrastructure, and management capacity. This aligns with participants’ emphasis on personalization and control. Moreover, given participants’ limited experience with ICT and struggles with voice user interface interactions shared in phase 1 focus groups [11], resident training sessions and ongoing technical support are required for successful implementation. These supports are inherently service-oriented, requiring dedicated staff, sustained resourcing, and the cultivation of trust with residents, and should be considered alongside, rather than assumed to be fully addressed by the technology itself.

In the future, research should examine how smart speaker–based social connection tools interact with other community resources and interventions. Our participants identified transportation barriers, lack of information about community activities, and limited social networks as interconnected challenges. Technology solutions are most likely to succeed when integrated into broader community-based approaches that address structural barriers to social connection.

Learning emerged as a prominent brainstorming category in phase 1, comparable in volume to Socializing With Others. Participants—all nonusers—generated ideas spanning news and current events, language acquisition, practical skills, and digital literacy. Although this domain falls outside the social connectedness focus of this study, these observations point to an interest in intellectual engagement within this population, consistent with a growing literature on lifelong learning in older adults [54,55]. Future work might more explicitly examine learning-oriented smart speaker–based applications as a complementary pathway for supporting cognitive engagement and quality of life.

### Limitations

There are several limitations of this study. First, due to the COVID-19 pandemic, we were unfortunately unable to follow up with some participants throughout the process, resulting in their loss from the study. The pandemic also necessitated off-site meetings with transportation provision, which may have excluded some potential participants with mobility limitations or discomfort with van travel. Second, established trust relationships that facilitated recruitment may have introduced response bias, as participants with prior positive experiences with the research team may have been more inclined to provide favorable feedback. Given that real-world implementation will inevitably encounter residents who are skeptical, mistrustful of institutional technology, or uninterested in smart speaker–based applications, purposive recruitment of such individuals would elicit insights that a self-selected, engaged sample cannot provide. Third, incorporating diverse and sometimes contrasting ideas into the design was challenging. While we used multiple data synthesis methods, a more systematic approach to identifying and prioritizing user demands (eg, conjoint analysis or formal prioritization exercises) would have been beneficial for making design trade-off decisions explicit. Fourth, hearing-related concerns, such as hearing loss that hinders meaningful engagement with smart speakers, did not emerge spontaneously in focus group discussions. This absence may reflect sample characteristics or participants’ limited awareness of the relationship between hearing ability and voice interface usability. Although smart speaker designs incorporate features such as adjustable volume and visual display companions that may support more equitable access, their adequacy in practice remains unclear. Future work should more explicitly examine how hearing loss shapes engagement with smart speakers. Fifth, while we adapted facilitation approaches to encourage broad participation, differential comfort with technology discussions likely shaped whose voices were most prominent in certain design decisions. Furthermore, differences in age, education, technology access and usage, and prior smart speaker experience among participants may have influenced the outcomes at each phase of the study. Finally, this study focused on early-stage prototyping and scenario development. The scenarios represent participant aspirations and preferences but have not been validated through implementation.

### Conclusions

Our study identified the needs for meaningful connections and safe, independent living among older adults living alone in affordable housing and showed the potential of smart speakers and their voice agents to address social isolation and reduce loneliness. Through a 3-stage iterative UCD process, we successfully engaged residents, including those with limited technology experience, in co-designing 7 prototype scenarios across 4 functional domains. The UCD methods used in this study effectively incorporated end user feedback to develop a technology platform for reducing social isolation and loneliness in this population [42]. Findings emphasize the importance of accessible, thoughtfully designed technology tools for social connectedness and more broadly for health and well-being tailored to older adults with limited resources and digital literacy. Further research is needed to fully develop these prototypes into functional applications, pilot-test them in real-world settings, and evaluate their effectiveness in addressing the unique social health needs of underserved older adults. Future evaluation should also extend beyond self-selected participants to deliberately engage skeptical residents, individuals with institutional mistrust, and those for whom this technology is not an intuitive fit. Rather than assuming universal applicability, research should iteratively test and refine these prototypes across diverse residential contexts, trust environments, and user dispositions to generate a more grounded and transparent evidence base to implementation in affordable housing communities.

## Supplementary material

10.2196/90053 Multimedia Appendix 1 Pictorial sheet for the 6-8-5 exercise for brainstorming in phase 1.

10.2196/90053 Multimedia Appendix 2 Social well-being questionnaire—social isolation, loneliness, and satisfaction.

10.2196/90053 Multimedia Appendix 3 Categories of ideas written or drawn on pictorial sheets.

10.2196/90053 Multimedia Appendix 4 Example ideas shared by a smart speaker user and nonuser.

10.2196/90053 Multimedia Appendix 5 Voice2Connect functional categories, description, and potential technology functions.

10.2196/90053 Multimedia Appendix 6 Initial use case scenarios.

10.2196/90053 Multimedia Appendix 7 A matrix of content analysis findings in phase 3.

## Abbreviations

AI: artificial intelligence
ICT: information and communication technology
UCD: user-centered design
