# Supplementary figures and images for: Smart Speaker–Based Applications to Support Social Connectedness in Older Adult Residents in Affordable Housing: User-Centered Design Study
Source: JMIR Aging. 2026 Jul 7;9:e90053. doi: 10.2196/90053 (PMC13340430; doi:10.2196/90053)

**Multimedia Appendix 1.**

Pictorial Sheet for the 6-8-5 Exercise for Brainstorming in Phase 1.

**
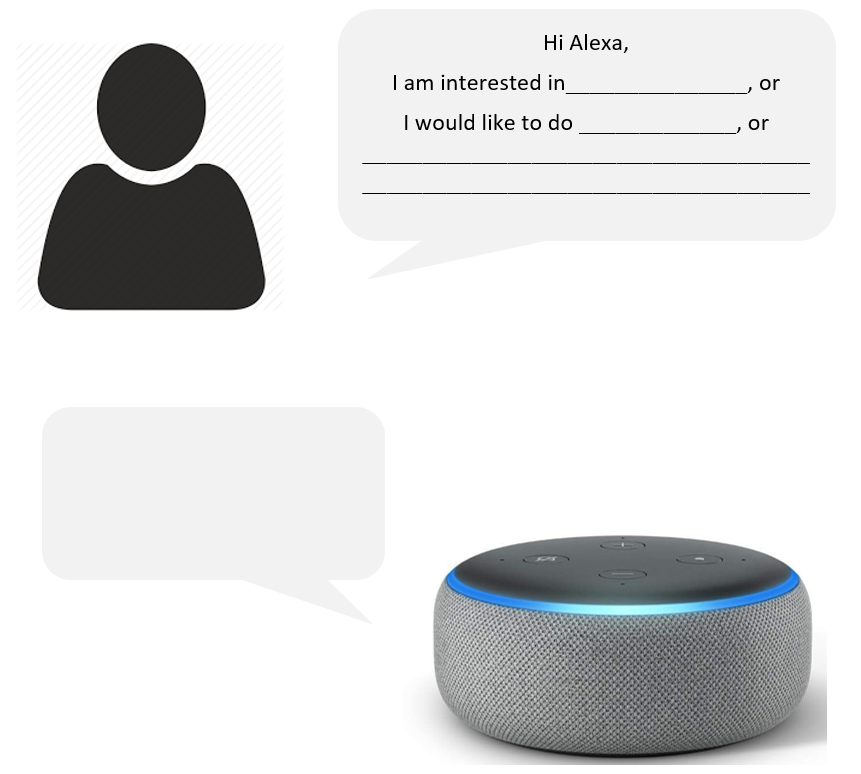
**

Supplement: Multimedia Appendix 1 [file aging-v9-e90053-s001.docx]
