# Supplementary material for: Smart Speaker–Based Applications to Support Social Connectedness in Older Adult Residents in Affordable Housing: User-Centered Design Study
Source: JMIR Aging. 2026 Jul 7;9:e90053. doi: 10.2196/90053 (PMC13340430; doi:10.2196/90053)
Supplement: Multimedia Appendix 2 [file aging-v9-e90053-s002.docx]

**Multimedia Appendix 2.**

Social Well-being Questionnaire - Social Isolation, Loneliness, and Satisfaction

The objective social isolation was measured by the Lubben Social Network Scale –

Revised (Lubben et al., 2006), consisting of six items that evaluate the size of older adults’ social networks – family/relatives and friends/neighbors. Total scores range from 0 to 30, with higher scores indicating larger social networks. Participants with a score of less than 12 were identified as socially isolated (Lubben et al., 2006; Rubinstein et al., 1994). Social activity participation was measured by one, yes or no question “Do you participate in any organizations, religious groups, or committees?”

We used the 3-item UCLA Loneliness Scale to assess loneliness. It consists of three items: “How often do you feel that you lack companionship?”, “How often do you feel left out?” and “How often do you feel isolated from others?” It uses a 3-point scale ranging from 1 (hardly ever) to 3 (often), with higher scores indicating greater loneliness. Participants with a score ≥ 6 were categorized as lonely (Saraiva et al., 2020).

Satisfaction with the current level of social interactions was measured by one item from the Duke Social Support Index (Koenig et al., 1993), “How satisfied are you with the relationships you have with your family and friends?” It had three response options: 1 = very satisfied, 2 = somewhat dissatisfied, and 3 = satisfied.

*References:*

Koenig, H. G., Westlund, R. E., George, L. K., Hughes, D. C., Blazer, D. G., & Hybels, C.

(1993). Abbreviating the Duke Social Support Index for Use in Chronically Ill Elderly

Individuals. *Psychosomatics, 34*(1), 61–69. https://doi.org/10.1016/S0033-3182(93)71928-3

Lubben, J., Blozik, E., Gillmann, G., Iliffe, S., Von Kruse, W. R., Beck, J. C., & Stuck, A. E.

(2006). Performance of an abbreviated version of the Lubben social network scale among

three European community-dwelling older adult populations. *Gerontologist, 46*(4), 503–513. https://doi.org/10.1093/geront/46.4.503

Rubinstein, R. L., Lubben, J. E., & Mintzer, J. E. (1994). Social isolation and social support: An

applied perspective. *Journal of Applied Gerontology, 13*(1), 58–72.

https://doi.org/10.1177/073346489401300105

Saraiva, M. D., Apolinario, D., Avelino-Silva, T. J., De Assis Moura Tavares, C., Gattás-

Vernaglia, I. F., Marques Fernandes, C., Rabelo, L. M., Tavares Fernandes Yamaguti, S.,

Karnakis, T., Kalil-Filho, R., Jacob-Filho, W., & Romero Aliberti, M. J. (2020). The Impact of Frailty on the Relationship between Life-Space Mobility and Quality of Life in Older Adults During the COVID-19 Pandemic. *Journal of Nutrition, Health and Aging, 25*(4), 440–447. https://doi.org/10.1007/s12603-020-1532-z
