# Supplementary material for: Smart Speaker–Based Applications to Support Social Connectedness in Older Adult Residents in Affordable Housing: User-Centered Design Study
Source: JMIR Aging. 2026 Jul 7;9:e90053. doi: 10.2196/90053 (PMC13340430; doi:10.2196/90053)
Supplement: Multimedia Appendix 3 [file aging-v9-e90053-s003.docx]

**Multimedia Appendix 3.**

Categories of Ideas Written or Drawn on Pictorial Sheets.

| **Category** | **The number of ideas** | **Example Ideas** |
| --- | --- | --- |
| Daily assistance | 41 | - Knowing if the elevator in the building is working - Control heat or air conditioning |
| Health and safety | 34 | - Call for help - Alexa telling me about my health if I’m feeling bad - Help me with methods to stay active in my mind - Call my doctor - Keep up with my blood pressure with a Fitbit watch or something else |
| Socializing with others | 26 | - Call my daughter - Talk to other residents - Have Alexa to call my son/friend so that I can know how they are, when I feel lonely and I don’t hear from my children |
| Learning | 28 | - Help in learning - Help to spell words - Computer tips |
| Companionship with the virtual agent Alexa | 9 | - Read a book to me - In the future, when I get really older and lonely, I say “Alexa, tell me good morning” - Alexa tells me I’m worth continuing to keep going and don’t give up |
| Entertainment | 7 | - Listening to music - Sports |
| Religion | 3 | - Tell me God loves me |
| Miscellaneous | 5 | - Cybersecurity - Lotto |
| **Total N** | **153** |  |
