# Supplementary material for: Smart Speaker–Based Applications to Support Social Connectedness in Older Adult Residents in Affordable Housing: User-Centered Design Study
Source: JMIR Aging. 2026 Jul 7;9:e90053. doi: 10.2196/90053 (PMC13340430; doi:10.2196/90053)

**Multimedia Appendix 4.**

Example Ideas Shared by a Smart Speaker User and Nonuser.

1. An idea from a smart speaker user

**
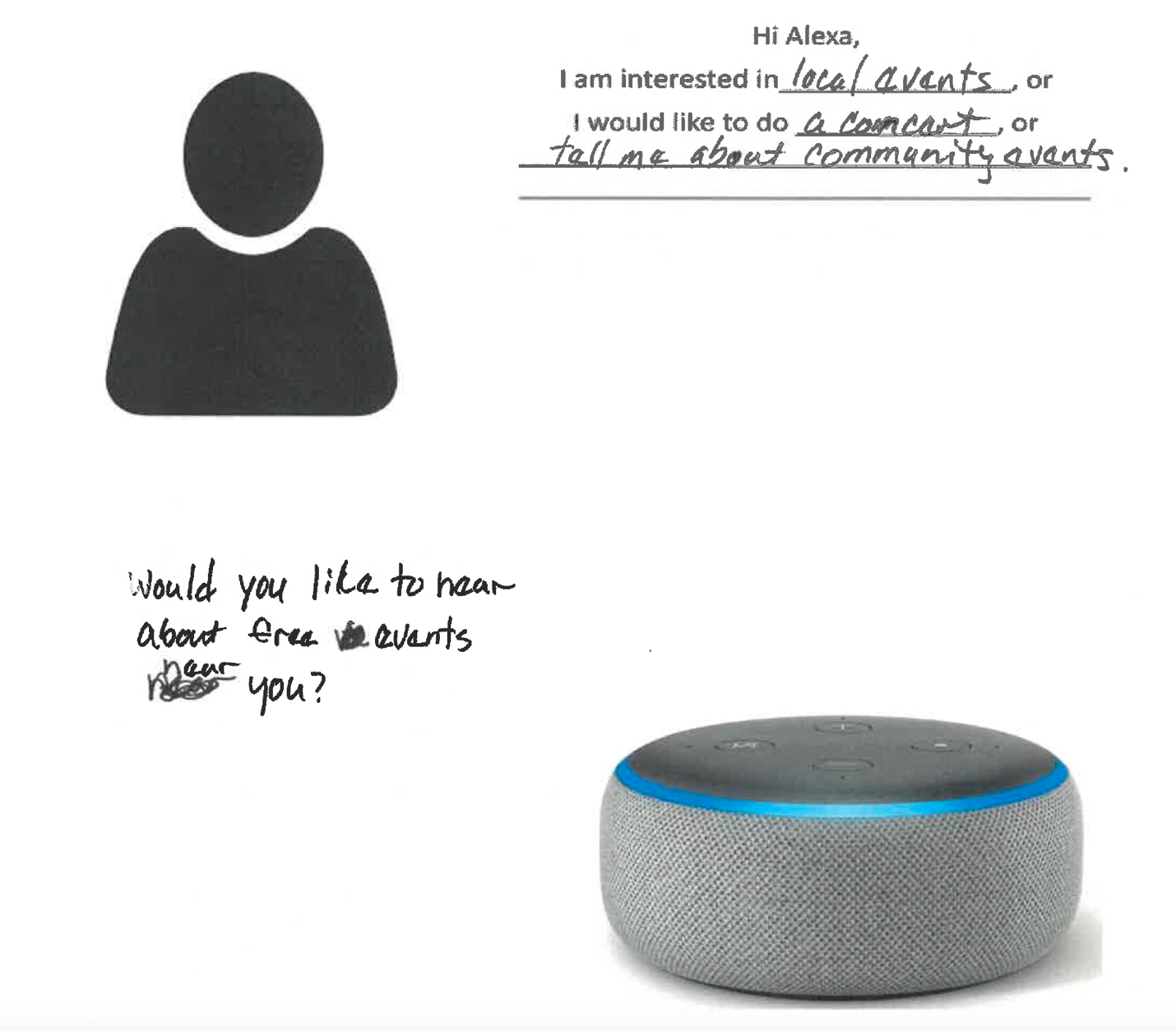
**

1. An idea from a smart speaker nonuser


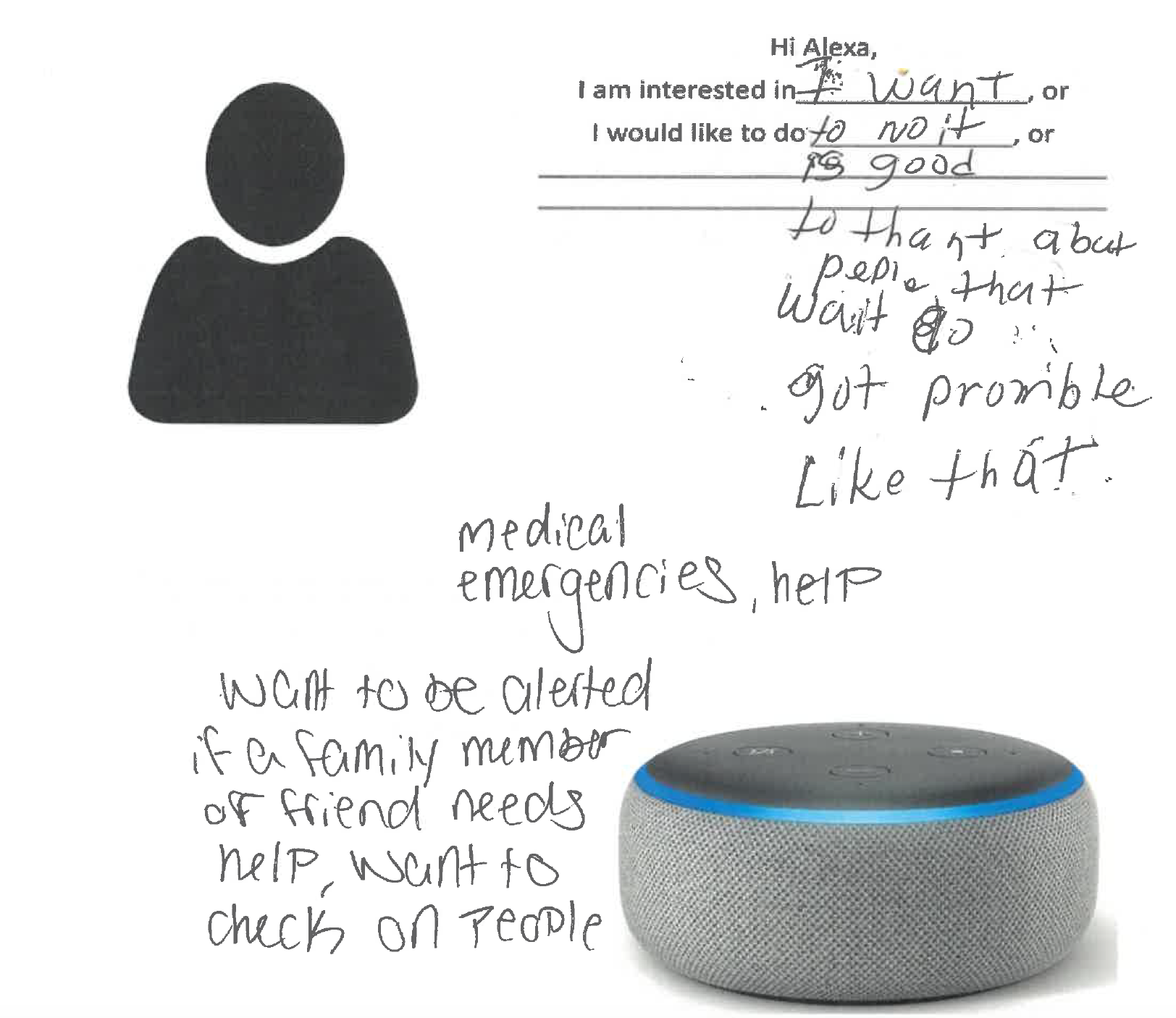

Supplement: Multimedia Appendix 4 [file aging-v9-e90053-s004.docx]
