# Supplementary material for: Smart Speaker–Based Applications to Support Social Connectedness in Older Adult Residents in Affordable Housing: User-Centered Design Study
Source: JMIR Aging. 2026 Jul 7;9:e90053. doi: 10.2196/90053 (PMC13340430; doi:10.2196/90053)
Supplement: Multimedia Appendix 5 [file aging-v9-e90053-s005.docx]

**Multimedia Appendix 5.**

Voice2Connect Functional Categories, Description, and Potential Technology Functions.

| Category | Description | Potential technology functions that can be incorporated into the technology design |
| --- | --- | --- |
| Checking in | This function focuses on assisting the user to check in on one another or assisting the housing management to check in on their residents to see they are well or to see if they are in any emergency situations. Also, the system could recognize that the user has not spoken to someone and remind them to keep track of whom to check on and how they’re doing. This function was deemed critical because all of the participants lived alone and voiced the need for someone to rely on in case of emergencies or unexpected health events. | Safety monitoring; emergency response systems; alert systems |
| Social companion | This function focuses on using the voice agent as a social companion. Residents could feel like they have somebody to talk to, or someone who enlightens their feelings to not feel lonely. A voice agent could also remember the previous conversation with the user and continue it. | Virtual social companion; remote intervention for emotional support |
| Community involvement | The technology could be used as a tool for enhancing social integration and connectivity in their housing community and a broader neighborhood. The technology could facilitate social participation by providing necessary information such as bus routes and times or enabling virtual meetings (e.g., church services). Also, it could be used as a tool for arranging meetups (both in person and online) or provide online chat rooms where residents could talk to other users (e.g., watching the same TV show and talking about it). | Event notification; information source for transportation access; reminders; interventions for social networks and support; online chat rooms |
| Wellness check | The technology could be integrated with health apps through wearables to monitor the user’s vital signs (e.g., blood pressure, heart rates, heart rate variability) and detect some issues related to health, wellness, and moods. It could be also used to recognize loneliness risks by looking at activity levels (e.g., increased indoor time) and intervene (e.g., message, an alarm to motivate physical exercise, meeting with others, going outside) | Monitoring of physiological functions and activity patterns; social isolation and loneliness risk mitigation by flagging changes in activity patterns; and social participation encouragement |
