# Supplementary material for: Smart Speaker–Based Applications to Support Social Connectedness in Older Adult Residents in Affordable Housing: User-Centered Design Study
Source: JMIR Aging. 2026 Jul 7;9:e90053. doi: 10.2196/90053 (PMC13340430; doi:10.2196/90053)
Supplement: Multimedia Appendix 6 [file aging-v9-e90053-s006.docx]

**Multimedia Appendix 6.**

Initial Use Case Scenarios. Italicized texts indicate the conversations between the user and Alexa.

| **Category 1: Checking-In**  Scenario 1: Using Alexa to talk to your neighbor/friend  Tom, 78-old male, uses the smart speaker to check in on others in the building. Tom hasn't heard or seen his neighbor Jeanne in two weeks, but he knows Jeanne also has an Alexa Dot, so he speaks to Alexa to see if Jeanne is doing well: *“Alexa, connect me to Jeanne”* and then he is connected to Jeanne. Tom asks Jeanne, *“Hey, how are you doing? I just want to make sure you are well.”* Tom and Jeanne chat briefly through the speaker to share what is going on with them. Two days after, the Alexa reminds Tom, *“Tom, you haven’t been in contact with Don for three weeks. Do you want to talk to him?”* Tom says *“Yes, connect me to Don. I want to see how he is doing.”*  Scenario 2: Check-ins from housing management  The office manager put an announcement on all of the Alexa’s in the building.  *“This is management.  Please tell Alexa if you are ok.  If we do not hear from you in 24 hours, we will come to your apartment to check on you.”* Cathy said, *“Alexa, tell management I am ok.  I have had a bad cold recently.”*  Lisa has been worried about her neighbor Cathy so she checks with management. Management tells Lisa that Cathy is ok and that she did not need to worry about her neighbor, she just has been sick in the past week. |
| --- |

| **Category 2: Social Companion**  Scenario 3: Being a friend with Alexa  George is a widowed 85-year-old male and lives alone. George’s children come and visit him on the weekends, but during the week he has little social interaction and often feels lonely.  Alexa detects when George gets out of bed and greets him by saying *“Good Morning, George, how did you sleep?”*  George replies saying, *“I slept well, Alexa, can you read me the next chapter of my Steven King book at 10 am?.*  At 10 am, Alexa says “*Here is where we left off reading yesterday”* and continues to read the book aloud to George.  After some time, George says, *“Alexa, stop reading.  This book is getting very good.”*  Alexa replies, *“It is.  What do you think is going to happen next?”*  George replies, and Alexa says, *“That is possible, we should read again tomorrow.”*  Scenario 4: Conversation between you and Alexa  John has arthritis in his knees and hips, making walking and standing for long amounts of time painful for him, so he likes to stay at home most of the time.  Getting out in the community and connecting with other people has been difficult because of John’s limited mobility. John likes to talk to Alexa.  In the mornings he will say *“Good morning, Alexa”,* and Alexa will reply, *“Good morning, John.  Today is going to be a blessed day.”*  John is able to reply, *“Every day is a blessed day.”*  John likes to talk out loud about what is happening in his favorite television show and movies.  Alexa remembers what John says is happening in the movie and is able to talk with him about it.  *“John, that is a popular movie. Online it has hundreds of 5-star reviews. One person left a review and said ‘this is one of my favorite movies. I was not a big fan of the ending, but overall the plot was good.”* John replies, *“Alexa, I agree, but it was still a good movie.”* |
| --- |

| **Category 3: Social Participation**  Scenario 5: Getting involved in the community  Alice, a 75-year-old female, recently moved into a new apartment building to be closer to her children and is having a difficult time getting settled in and meeting new friends in the building.  Alexa detects that Alice has not left her apartment in over a week.  Alexa says *“Alice, your neighbor, Martha, is going for a walk this afternoon and is looking for someone to join her, would you like me to connect you to Martha so you can communicate with her about the afternoon walk?”*  On Sunday, Alexa says to Alice *“Good morning Alice, there is the weekly church service going on in the community room downstairs.  Everyone in the building is invited.”*  Scenario 6: Arranging meet-ups  David is a new resident of affordable senior housing and recently got an Alexa.  After settling into his new apartment, Louise is connected to David’s Alexa and says *“Hi David, my name is Louise, a small group is getting together downstairs to meet all the new residents that moved in this month.  We would love to meet you.”*  A couple of days later, David made a list of grocery items he needed.  Being new to Richmond, David did not feel comfortable going alone.  He said *“Alexa, can you ask if anyone needs to go to Kroger today?  We can all take the bus together.”*  Alexa replies*, “Martha will go to Kroger with you today at 2 pm.  You can take the #3 bus at 1:30. It will pick you up in front of the building.”* |
| --- |

| **Category 4: Wellness Check**  Scenario 7: Wellness and Mood Check  Paula, a 69-year-old female, has been living in her apartment building for four years.  She would describe herself as fairly active and social with a few good friends living in the same apartment building.  Recently, her only child and grandchildren moved further away from Paula, making it more difficult to see each other.  Since then, Alexa detects that Paula is seeing her friends less frequently, sleeping in later, taking naps, and is spending more time watching TV.  Alexa says *“Paula, the data from the Fitbit tells that you have not been as active as you normally are.  How are you doing? Would you like me to connect you to a doctor?”*  Paula says “*Ok, Alexa, schedule me a doctor’s appointment.”*  Three days later, with an upcoming doctor’s appointment, Paula still has not left her apartment. Alexa *says “Paula, your doctor’s appointment is next week.  Perhaps in the meantime, I can connect you with your friend Stephanie to go for a walk together.”* |
| --- |
