# Supplementary material for: Smart Speaker–Based Applications to Support Social Connectedness in Older Adult Residents in Affordable Housing: User-Centered Design Study
Source: JMIR Aging. 2026 Jul 7;9:e90053. doi: 10.2196/90053 (PMC13340430; doi:10.2196/90053)
Supplement: Multimedia Appendix 7 [file aging-v9-e90053-s007.docx]

**Multimedia Appendix 7.**

A Matrix of Content Analysis Findings in Phase 3.

| **Scenario** | **Likes** | **Concerns** | **Recommendations** |
| --- | --- | --- | --- |
| **Category 1: Checking-In** | | | |
| #1: Using Alexa to talk to your neighbor/friend | - The scenario presented a convenient way of connecting compared to phones or in-person check in. - It could be another source of technology to communicate with people who are in need and who do not have family nearby. - It would make them feel more connected. | - Information about a resident’s status should not be shared with anyone. | - Add the ability to control and monitor who checks in on me. - Allow for a ‘close friends list’ or ‘contact list.’ - Add a do-not-disturb feature or a Voicemail which allows residents to choose if they want to answer someone checking in on them. - A video interface would be helpful to remove accessibility barriers (e.g., visual or hearing impairments) to connecting to people. |
| #2: Check-ins from housing management | - It would be great to check in on especially residents with mobility disability or who don’t come out of their apartments. - This seemed like an easy way to stay up to date with management and events in their housing. | - Housing management is sometimes unreliable and cannot always be trusted. - Management should not dive too deep into their residents’ lives. Their role should be limited to just checking in. - It would be challenging to keep the information feeding into the management side up to date. | - Add the ability to select when to be checked on by others. - Add information about community-based events (e.g., classes in the housing, community meals) to the types of management announcements. |
| **Category 2: Social Companion** | | | |
| #3: Being a friend with Alexa | - The idea of Alexa talking to a user and providing news updates could help people feel engaged and connected, contributing to a sense of alertness while keeping them mentally and emotionally active. - It could help people who feel lonely and lack assistance. | - There was a skepticism about where the device gets the information to maintain a conversation. - There was a fear of losing human contacts. - It felt unnatural to talk to a voice agent as if it were a human. - It should be personalized to user needs. | - Allow for this feature to be turned on and off so that residents have a choice whether or not they want to use it. - Give Alexa the ability to answer questions more deeply and more "human like" than it can currently. |
| #4: Conversations between you and Alexa | - Giving lonely people some type of artificial companionship is a good idea. It could help lonely people. - Non-users seemed to be open to the concept and recognized that it could be useful for the future. | - There was an expressed need by some participants to talk to a human being instead of an artificial intelligence-based agent even though they felt it is better than nothing. - Mechanical voices and the repetitiveness nature would make people bored. | - Allow for this feature to be turned on and off so that residents have a choice whether or not they want to use it. - Give Alexa the ability to answer questions more deeply and more "human like" than it can currently. |
| **Category 3: Community Involvement** | | | |
| #5: Getting involved in the community | - It would help connect with people. - Advertising community events would promote social interactions among residents and help build a sense of community. | - There is a need to have face to face interactions before allowing the system to broadcast to build trust. | - Create a virtual bulletin board so that residents know what is happening in their building and a broader community. - Simplify steps by removing the need to go through another application. |
| #6: Arranging meetups | - It could help connect with others to do something together or with someone who has knowledge you don’t have (e.g., catching the bus). - It would be a convenient tool for individuals with limited mobility. - Automatic arrangements of going out for necessity was well received by some participants (e.g., going to a grocery store together). - It would provide an opportunity to meet different people. | - There was a privacy concern about unintentional sharing of information about their whereabouts and what they are doing. | - Create a virtual bulletin board so that residents know what is happening in their building and a broader community. - Create two-level notifications: 1) building-level event announcements and 2) friend-to-friend notices. - Add a close contact list for arranging meetups. |
| **Category 4: Wellness Check** | | | |
| #7: Wellness and Mood check | - It could help individuals who are isolated and need to be motivated out of their isolation. | - Some felt this function would be an invasion of privacy if it were designed to detect an emotional status. - There was a preference for the voice agent to not start talking out loud unexpectedly. - There was a skepticism about the feature of monitoring behavior metrics. | - Allow an option to choose which physiologic and activity metrics need to be monitored. - Make the voice agent respond only to the user’ voice. |
